# Charting the contributions of cognitive flexibility to creativity: Self-guided transitions as a process-based index of creativity-related adaptivity

**DOI:** 10.1371/journal.pone.0234473

**Published:** 2020-06-11

**Authors:** Yihan Wu, Wilma Koutstaal

**Affiliations:** 1 Graduate Program in Cognitive Science, University of Minnesota, Minneapolis, Minnesota, United States of America; 2 Department of Psychology, University of Minnesota, Minneapolis, Minnesota, United States of America; University of Bologna, ITALY

## Abstract

Creativity is pivotal to solving complex problems of many kinds, yet how cognitive flexibility dynamically supports creative processes is largely unexplored. Despite being a crucial multi-faceted contributor in creative thinking, cognitive flexibility, as typically assessed, does not fully capture how people adaptively shift between varying or persisting in their current problem-solving efforts. To fill this theoretical and methodological gap, we introduce a new operationalization of cognitive flexibility: the process-based Self-Guided Transition (SGT) measures, which assess when participants autonomously choose to continue working on one of two concurrently presented items (dwell length) and how often they choose to switch between the two items (shift count). We examine how these measures correlate with three diverse creativity tasks, and with creative performance on a more complex "garden design" task. Analyses of the relations between these new cognitive flexibility measures in 66 young adults revealed that SGT dwell length positively correlated with creative performance across several tasks. The SGT shift count positively correlated with within-task performance for a two-item choice task tapping divergent thinking (Alternative Uses Task) but not for a two-item choice task calling on convergent thinking (Anagram task). Multiple regression analyses revealed that, taken together, *both* the shift count and dwell length measures from the Alternative Uses Task explained a significant proportion of variance in measures of fluency, and originality, on a composite measure of the three independently-assessed creative tasks. Relations of SGTs to the Garden Design task were weaker, though shift count on the Alternative Uses Task was predictive of a composite measure of overall Garden Design quality. Taken together, these results highlight the promise of our new process-based measures to better chart the dynamically flexible processes supporting creative thinking and action.

## Introduction

Adaptive creative problem-solving requires dynamic integration of multiple sources of motivational, cognitive, and perceptual information relating to our goals and task progress. In complex creative problem-solving, it is crucial at times to be persistent and at other times to be flexible depending on the task requirements, our task goals, and our progress, or perceived progress, toward those goals [1–3]. Yet little is known about how we dynamically exercise the flexible cognitive control that allows us to adaptively shift from doggedly pursuing our current path to newly exploring alternative routes, and how this shapes our creative thinking and action.

The aim of the current research is to examine how cognitive flexibility contributes to varied conceptual and perceptual creative task performance, using new proposed measures to more naturalistically operationalize the construct of cognitive flexibility and creativity-related adaptivity. The measures assess how participants freely choose to allocate their efforts on one of two related problems across time (Self-Guided Transitions) in an idea generation task and an orthographic recombination task.

Contextually-modulated behavioral transitions during creative tasks have been shown to positively correlate with creative performance using think-aloud protocols in ecologically complex creative design tasks [4] and in cognitive control tasks such as the color-word Stroop task [3]. For example, during complex creative design tasks, experienced designers have been found to continually move between different subgoals as they iteratively and progressively discover, define, and seek to address emerging design issues or opportunities [5–7], and the frequency of such within-task transitions increases both with greater expertise, and leads to higher quality design outcomes [4]. Within experimental psychology, questions relating to cognitive flexibility have been extensively examined using experimenter-cued task-switching paradigms (e.g., number-letter tasks) [1, 8] and set-shifting tasks such as the Wisconsin Card Sorting Task [9] and related tasks [10]. One robust finding from experimenter-cued task-switching paradigms is that participants are slower on the trials that they are cued to switch than on the repeated trials [11]. Nonetheless, in all of these tasks, to allow for maximal experimental control, the problem space is narrowly defined and tightly structured, with alternative task stimuli or task rules that would not typically be juxtaposed in daily tasks. These tasks thus largely lack generalizability to everyday complex problem solving.

Researchers also have adapted the experimenter-cued task switching paradigm to assess voluntary task switching where, for example, participants are instructed to switch randomly but equally often between two designated tasks [12, 13]. Illustrative findings from this paradigm are that participants show lower error rates on voluntary-switch compared with forced-switch trials [14, 15], and that the expectation of increasing reward is associated with a higher switching rate [15]. These findings highlight that different cognitive-motivational processes may be invoked when individuals have a choice regarding the task content rather than attempting to follow externally-imposed cues for when to switch. However, similar to experimenter-cued paradigms, most voluntary task-switching studies also use simple stimuli and arbitrarily juxtaposed tasks, with different goals and/or rules for each task. More importantly, an individual's natural tendency to shift or to dwell has rarely been tested [16], and thus it is hard to link to an individual's spontaneous tendency to work on a different problem or different problem aspect in everyday life.

More naturalistic and less-constrained tasks have been used in classic neuropsychology to assess spontaneous flexibility rather than reactive flexibility [17], using tests such as semantic fluency (e.g., generating the names of animals) and phonemic fluency (e.g., generating English words beginning with the letters F, A, or S). For such tasks, investigators have derived measures of clustering and switching based on the semantic and/or phonological similarity of successively generated responses [18]. Findings from these paradigms show that both shifting and clustering contribute to verbal fluency, with shifting especially calling on cognitive control processes [19] whereas clustering is more strongly associated with automatic processes, such as automatic semantic and lexical associative links between words [20, 21]. Investigators have also examined the time required to move within and between clusters of responses on divergent thinking tasks such as a version of the Alternative Uses Task, finding that the latency of consecutive responses was longer when participants switched to a different category than when they stayed within a given category [e.g., 22]. Nonetheless, despite the insights that measures of clustering and switching have provided, these measures are entirely dependent on the responses produced by the participants. The clusters and switches must be inferred and it can be unclear whether the inferred clusters match the mental categories actually held by the participants. Furthermore, clustering and switching assessments have not allowed the temporally-tied observation of the dynamic cognitive processes and participant-initiated allocation of effort involved in choosing between alternative task items and producing the content.

Here, we provide participants opportunities to freely choose how to allocate their efforts to one of two closely related problems across time [cf. 23, 24] with identical goals and/or rules for each task. We assess how often participants "shift" to working on the second problem versus "dwell" in solving the current problem, thereby obtaining indices that are separable from the specific content of their responses. Movement between the two task items is thus conceptually similar to movements between different "patches" in information-foraging paradigms [e.g., 25]. To evaluate the generality of self-guided switching/dwelling (Self-Guided Transitions, or SGTs) in two different contexts, we employed both a widely used conceptual-perceptual measure of divergent thinking (the Alternative Uses Task) and a convergent orthographic recombination task (Anagram solving). Each task was initially administered in a single-item format (to acclimate participants to the task and task requirements) and then in a two-item format (two-item AUT and two-item Anagram), during which we extracted the number of participants' self-guided switches between the two items (switch count) and the number of responses they, on average, generated during each time they continued working on a given item (dwell length).

Self-guided switching that is closely attuned to task-difficulty and task-performance could arise from broad integration of cognitive, perceptual, and motivational/goal information [10, 26, 27] in turn benefitting complex and creative problem-solving [28–30]. To evaluate the generality of the association between cognitive flexibility and creativity we assessed creative performance with a diverse set of conceptually-based and perceptually-based creativity tasks, as well as with both outcome and process measures for a more complex and "naturalistic" Garden Design task, that was administered with a think-aloud protocol and in which participants provided concurrent sketches of their evolving garden [31]. We included both commonly used lab-based creative performance measures, such as the Suppose subtest of the Torrance Tests of Creative Thinking [32], and novel perceptually-and-conceptually-based measures assessing varied interpretation of ambiguous figures (Figural Interpretation Quest, Koutstaal & Tran, in prep.) and concept word-pairs or conceptual-combination. Inclusion of a diverse set of creativity tasks and creative performance aspects (e.g., fluency and originality) together with the more complex naturalistic Garden Design task provided a unique opportunity to examine the relative predictive value of Self-Guided Transitions across various creative tasks and performance aspects. It also, for the first time, allowed examination of whether self-guided transitions in a more structured "two-item" task context correlated not only with the novelty or originality of responses on the *same task* on which between-item shifts were assessed [23, 24], but also to creativity (fluency and originality) across *different domains* of assessment, separating out the within-task effects of transitions on creative performance from across-task effects.

We hypothesized that self-guided transitions would predominantly reflect participants' receptive attunement to their own unfolding progress toward their task goals [26, 29, 33, 34], signaling whether they should persist in their current direction (that is, continue working on the same item), or instead take an alternative route, indicative of flexible cognitive control, and so would be beneficial to creative performance. Prior research suggested some specific hypotheses for within-task SGT measures in relation to creative originality. Specifically, previous findings showed that more frequent (externally prompted) within-task shifting between two items was associated with higher novelty/originality of responses on the AUT [23] and also greater novelty of responses on two different category generation tasks [24] that required flexible searching of semantic/episodic memory (generation of "sense impression" categories, such as cold things and heavy things, and generation of items in *"ad hoc"* goal-related categories, such as things to take camping and fattening foods). Therefore, we predicted that self-guided within-task shifting (AUT shift count) would be significantly positively correlated with AUT originality. It was not clear if within-task AUT shift count would correlate with fluency, as these previous studies [23, 24] found mixed results for more frequent externally-guided shift effects on fluency. For the two-item anagram task, newly used here, and which comprised a tightly constrained stimulus-limited generation task (each item set had nine letters and participants were asked to generate words of four letters or more), our predictions for shift count and dwell length were less clear. However, previous partially-related research with verbal and phonemic fluency tasks has shown beneficial effects of both shifting and clustering on fluency [21], suggesting that our within-task measures of shift-count and dwell-length might positively correlate with the within-task measure of the number of words generated (analogous to fluency) for the anagram task.

Given that prior research on shifting in relation to indices of creativity (e.g., originality) has only examined within-task measures of shifting [23, 24] or transitions within a more-extended complex task relative to the quality of creative performance on the same task [4], there were few existing empirical findings to constrain our hypotheses with regard to how measures of shift-count and dwell-length on one task (e.g., the two-item AUT) would relate to assessments of creativity *across-tasks* (e.g., originality on the Torrance Suppose, Conceptual Combination, or Figural Interpretation tasks). However, based on growing empirical findings and theoretical accounts demonstrating that creative performance is associated with the interlinked contributions of both flexibility and persistence [2, 35], and divergent and convergent thinking [5], we hypothesized that both shifting and dwelling together would predict creativity across tasks. We assessed how well the combined self-guided transition measures from each two-item task (shift-count and dwell-length from AUT, and shift-count and dwell-length from Anagram) predicted overall originality and overall fluency scores (across all of the other creative tasks), and also how well the SGTs predicted overall quality of performance on the Garden Design task.

## Methods

### Participants

Participants (N = 81, 58 female, 23 male) were undergraduate students (average age 20.11 years, SD = 2.29). All participants were required to meet the criterion of being self-reported native speakers of English, between the ages of 18 and 30 years, and having normal or corrected-to-normal vision and hearing. They took part in return for research participation credits. Due to technical difficulties at the outset of the study in obtaining and saving the SGT digital screen recordings, we obtained SGT data for 66 participants tested after our recording set-up was finalized, including all but one participant who was inadvertently not screen-recorded. Accordingly, all of the analyses for the SGTs are based on N = 66 (46 female, 20 male, M age = 21, SD = 3.21) with the exception that the think-aloud for the Garden Design Task was not audio-recorded for one participant. To ensure consistency in the administration of the think-aloud protocol for the Garden Design Task, one experimenter conducted all testing sessions. Although given that we were using newly developed measures, there was little information to guide an a priori power analysis, we aimed to acquire a sample size similar to, or larger than, comparable studies in the literature [3, 4, 24]. For example, Atman et al. [4] included 24 and 26 participants per condition, and Smith et al. [24] included between 12 and 30 participants per condition. Thus, given our entirely within-subject correlational design, our sample size was more than double that of those prior related studies.

### Experimental design

This was a within-subject design, in which the participants were administered a series of perceptual and conceptual tasks of which we here report: (1) Alternative Uses Task (AUT), (2) Anagram task, (3) Garden Design, (4) Conceptual Combination (CC), (5) Figural Interpretation Quest (FIQ), (6) Torrance Consequences (“Suppose”) subtasks. To acclimate participants to the AUT and Anagram tasks, they were initially given one item to work on, followed by the two-item set with different stimuli. The tasks were administered in the order listed above, interspersed with a short break, followed by two brief metacognitive questionnaires that retrospectively probed the potential motivational impetus for participants' choices to switch or to dwell (further described under Dependent Measures) during the two-item AUT and two-item Anagram tasks. Table 1 provides examples of the task stimuli, and presentation details.

**Table 1:** Example stimuli for the six tasks, including presentation details.

Task	Examples	Number of Items	Duration
Alternative Uses Task (AUT)	One-set: Cup Two-set: Blanket, Flashlight	1 2	3 minutes 6 minutes
Anagram	One-set: pwiorekay Two-set: bfiojerac pvioxelam	1 2	3 minutes 6 minutes
Conceptual Combination (CC)	*What are the various things this combination of words could mean*? waterfall–jacket	3	3 minutes per item
Figural Interpretation Quest (FIQ)	*What various things could this object be*? (an ambiguous shape is shown to participants)	6	40 seconds per item
Torrance Suppose Task ^a^	*Just suppose you could become invisible*. *What interesting things might result*?	2	5 minutes per item
Garden Design Task	*Draw an initial plan for the design of a garden that is based on “a journey and the series of experiences those who walk around the garden will have on this journey”* (adapted from Pringle & Sowden, 2017).	1 (plus 1 practice item)	15 minutes

^*a*^ The example displayed for the Torrance Suppose Task is not one of the actual stimuli but is presented for illustrative purposes.

The Garden Design Task was closely modeled on the task developed by Pringle and Sowden [31]. Before the task, participants were given a think-aloud practice item and think-aloud instructions, during which they were asked to verbally "think aloud" while drawing a picture of an open book on a table. The task was administered in line with recommended procedures for administering think-aloud protocols as a method to allow observation of internal thinking processes without strongly altering participants' cognitive processes [31, 36]. Specifically, participants were instructed to "speak out whatever you are thinking at the time," "don't worry about complete sentences," and "don't over explain or justify." Participants were encouraged to speak as freely and continuously as possible. Feedback to prompt participants to follow these instructions was provided during the think-aloud practice task. Then participants were instructed to sketch the design of a garden based on “a journey and the series of experiences those who walk around the garden will have on this journey.” Participants were given design constraints relating to the budget and scale of the garden. They were encouraged to be as creative as possible, and to provide labels and a title for their design. Participants were asked to think-aloud throughout the 15-minute task duration.

### Stimuli

#### Procedure

Participants were tested individually in a single experimental session that began with obtaining written informed consent. At the conclusion of the experiment, participants were thanked, debriefed, and compensated. The study was approved by the University of Minnesota Institutional Review Board.

#### Process measures

**Fig 1:**
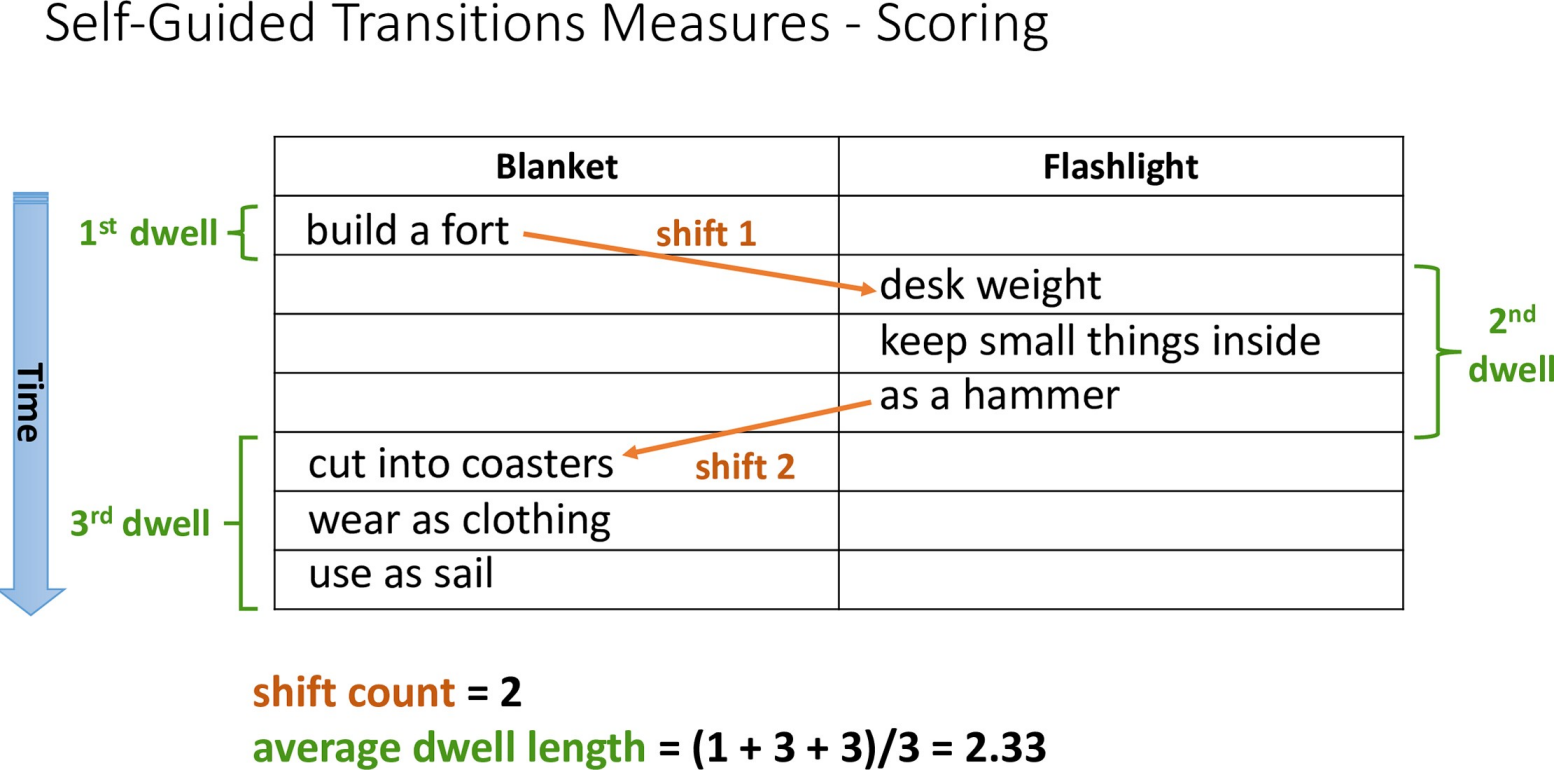
Demonstration of self-guided transition calculations (shift count and dwell length).

**Self-guided transitions.** To assess Self-Guided Transitions, a computer-screen recording tracked which item the participants were working on at any given time allowing us to retrospectively assess how often they switched from one of the two items to the other (switch count), and also how many solutions they generated for each item before they switched away (dwell length). Fig 1 illustrates how the shift count and average dwell length would be calculated for a subset of example AUT responses.

As shown in Fig 1, shift count was calculated as the number of times a participant chose to work on a task item different than their current one. For example, in the illustration, the participant shifted from initially working on "Blanket," to working on "Flashlight," and then back to "Blanket," resulting in a shift count of 2. Dwell length was based on the number of responses that a participant consecutively provided before they shifted to work on a different task item. Specifically, the number of responses they generated before alternation was counted from the screen recordings, and then summed and divided by the number of times they worked on either of the two task items, to obtain their average dwell length score. For instance, as shown in Fig 1, the participant initially generated one response for the task item "Blanket," then shifted to work on the task item "Flashlight" for three responses, and then shifted back to "Blanket," and generated three responses. Thus, their average dwell length score was (1 + 3 + 3) / 3 = 2.33. Note that this scoring approach takes into account all responses of the participants, including occasions where the participant provides only one response before they switch (see S3 Data). This approach to scoring tracks a participant's progress continuously, without omitting any responses, thereby capturing all of their information-foraging actions [25], and so reflects the full dynamics of their thinking process.

#### Dependent measures

**Creativity performance: Lab-based tasks.** As in many studies of creativity, participants' responses to the four lab-based creativity tasks were evaluated for different aspects, including fluency, originality, and flexibility [e.g., 37]. Adopting a rater-based scoring approach [e.g., 3, 38, for review see 39] each of the creativity tasks was scored anonymously by two independent raters blind to our hypotheses. The raters were first given scoring manuals with detailed scoring rubrics and were trained to reach acceptable levels of inter-rater reliability. Raters evaluated each response individually. Table 2 presents the inter-rater reliability for the four creativity tasks.

**Table 2:** Inter-rater reliability for the four lab-based creativity tasks.

Measures	Inter-rater reliability
Alternative Uses Task	
fluency	1.00
originality	0.97
semantic flexibility	0.87
reconstructive flexibility	0.80
Figural Interpretation Quest	
fluency	0.99
flexibility	0.78
originality	0.79
Concept Combination	
fluency	0.93
originality	0.81
Suppose	
fluency	0.99
originality	0.84

The AUT responses were scored for fluency (number of valid responses), originality (scored as 0, 1, or 2, with 2 representing highly unique or innovative responses, and summed), semantic flexibility, and reconstructive flexibility. Semantic flexibility was based on the number of different semantic categories (e.g., buildings, or education) into which responses could be classified, based on the 28 categories defined in the commonly used Torrance scoring manual. Reconstructive flexibility was based on the number out of 8 possible reconstructive categories that participants' responses involved, such as alternative uses based on object shape, object parts, adding motion, or material quality. The reconstructive flexibility measure was developed here to capture variation in how participants thought about the common object or their possible perceptual-motor interactions with the object. The FIQ task was scored for fluency (number of valid responses), category flexibility (number of different semantic categories out of a specified list of categories such as plants or trees, buildings, or vehicles), and originality (scored as 0, 1, or 2, with 2 representing highly unique or innovative responses, and summed). The Conceptual Combination task responses were scored for fluency (scored as 0, 1, or 2, with scores of 1 and 2 given for responses that incorporated one or both of the two concepts respectively), and originality (scored as 0, 1, or 2). The Torrance Suppose Task was scored for fluency (number of valid responses) and originality (based on the Torrance scoring manual designating "zero-originality" responses). For each of the creativity tasks, the assignment of zero for nonoriginal responses limited the coupling of fluency and originality because nonoriginal responses received no credit. The scoring scale of 1 or 2 for the originality of each specific response allowed raters to differentiate between responses that were somewhat original versus substantially original, without making unnecessarily fine-grained and unstable distinctions [40].

**Creativity performance: Garden design task.** Participants' think-aloud responses during the Garden Design Task were audio-recorded, transcribed, separated into idea units (distinct thoughts), and then coded for both the content of their verbalizations (e.g., references to plants, or activities such as resting) and their cognitive processes (e.g., idea generation vs. idea evaluation). For the Garden Design task, the average interrater reliability across the 7 sketch scores was .78, so we also used the average of the two raters' scores. Two raters separately coded the Garden Design think-aloud transcripts, with both raters coding 22.5% of the transcripts. They achieved interrater reliability of .87; for the transcript measures, the scores are based on one of the two raters (see Transcript content scores in the section on the Garden Design Task and SGTs, and S1 Data). The frequency with which participants moved between thinking phases of idea generation versus idea evaluation was also tabulated (Garden shift), as well as the average number of ideas they generated before shifting to the other thinking mode (Garden dwell ideation and Garden dwell evaluation); see Transcript transition scores in the section on the Garden Design Task and SGTs, and detailed description in S1 Data. Additionally, participants' sketches for the garden were separately assessed on several dimensions (e.g., journey diversity, structure; see S1 Data). Then, to obtain a comprehensive composite measure of the overall quality of participants' performance on the Garden Design task, we combined the seven sketch scores (journey diversity, originality, structure, elaboration, abstraction, scale, and budget) and three transcript content scores (idea unit count, idea category sum, and category sum). The last three scores represented, respectively, the number of distinct thoughts, the sum of ideas across different categories, and how many of the categories the transcript covered out of 218 possible categories. We first z-scored each specific measure, and then combined them into a composite assessment of overall quality on the Garden Design task, referred to as "Garden Quality10".

**Metacognitive questionnaire.** We developed two 9-item questionnaires on which participants were asked to retrospectively indicate the extent to which different cognitive and motivational factors contributed to their choices to switch or to dwell on the two-item AUT and two-item Anagram tasks (see S2 Data). Based on the pairwise correlations and exploratory factor analyses of responses to each questionnaire, the 9 items in each questionnaire were combined into 7 subscales. The 7 subscales were: (1) how enjoyable/challenging they found the two-set compared with the one-set task *(Enjoy/Challenge*, 2-items, challenge reverse scored); (2) the extent to which they chose to stay with one set, or to switch to the other set, based on how easy they found generating responses (*Choose dwell for easier*, 1 item); (3) the extent to which they noticed they were switching during the two-set task (*Notice switch*, 1 item); (4) the extent to which they intentionally switched during the two-set task (*Intentionally switched*, 1 item); (5) the extent to which they switched when they were stuck (*Switch when stuck*, 1 item); (5) the extent to which they switched when they wanted to work on something different or new (*Switch for new*, 1 item); and (6) the extent to which they found switching was helpful, or interruptive, in thinking of solutions (*Switch helped*, 2 items, interruptive reverse scored). All of the items were answered on a 5-point Likert-scale (1 = Strongly disagree, 2 = Disagree, 3 = Neutral, 4 = Agree, 5 = Strongly agree); additionally, participants were given the option of indicating that the item was not applicable (6 = Not applicable). Answers of "Not applicable" were coded as no response. Items were z-scored for further analyses.

## Results

### Descriptive statistics and correlations among SGTs

All participants' data passed validity and outlier screening checks. As reported earlier in the Method section, the interrater reliability for the lab-based creativity tasks and Garden Design scores was acceptable to excellent. Table 3 gives descriptive statistics for all of the lab-based measures.

**Table 3:** Descriptive statistics for all lab-based measures.

Type of Measure	Task	Mean	SD
Performance Measures	AUT Fluency	6.96	2.47
	AUT Originality	7.92	3.28
	AUT Semantic Flexibility	4.42	1.39
	AUT Reconstructive Flexibility	3.06	0.85
	Anagram Correct	8.28	3.13
	Anagram Letter Count	7.65	0.91
	Anagram Word Length	1.46	0.37
	CC Fluency	11.21	3.45
	CC Originality	2.68	1.55
	FIQ Fluency	4.41	1.01
	FIQ Category Flexibility	3.52	0.66
	FIQ Originality	0.45	0.15
	Suppose Fluency	12.16	3.66
	Suppose Originality	2.33	1.53
Process Measures	AUT Shift	5.12	3.34
	AUT Dwell	2.86	1.80
	Anagram Shift	4.35	3.30
	Anagram Dwell	4.46	2.63

AUT = Alternative Uses Task; CC = Conceptual Combination; FIQ = Figural Interpretation Quest. The values reported for the AUT and Anagram are based on participants' responses during the respective two-item tasks. Although not of central interest, we also here report the number of correct responses for the Anagram task, as well as how many of the 8 possible letters were used, and variation in the word length of correct responses (assessed as the range between the shortest and the longest correct responses).

Table 4 gives the across-task correlations for the new self-guided transition measures. Within the AUT or Anagram tasks, how were shift count and dwell length related to one another? And did participants who relatively frequently switched from one task item to the other task item on the loosely structured AUT, show a similar tendency on the more structured Anagram task? Paralleling findings from many analogous studies that used content-based (inferred) measures of shifting and clustering/dwelling, it can be seen from Table 4 that, within the AUT task, there is a moderately strong negative correlation (*r* = –.58) between the shift count and dwell length measures. A similar negative correlation (*r* = –.58) is found between the shift count and dwell length measures for the Anagram task. The magnitude and direction of these correlations indicates that although shifting and dwelling were inversely related to one another, the shift count and dwell length measures nonetheless provide substantial nonoverlapping information. A different pattern was observed when looking at individuals' tendencies to shift or to dwell across the two tasks. Whereas participants' shifting behavior was largely uncorrelated across the AUT and Anagram tasks, the dwell measures were positively correlated across these two quite different tasks (*r* = .31).

**Table 4:** Correlations among the self-guided transition measures.

Flexibility Measure	1	2	3	4
1. AUT shift	--			
2. AUT dwell	-.58**	--		
3. Anagram shift	.11	-.18	--	
4. Anagram dwell	-.19	.31*	-.58**	--

** *p* < 0.01 (2-tailed)

* *p* < 0.05.

In an exploratory vein, we also examined, for the conceptually-based AUT divergent thinking task, how our new process-based assessments of cognitive flexibility related to commonly used content-based semantic flexibility scoring (see S3 Data). Specifically, we obtained several content-based measures of semantic category clustering and shifting both for the single AUT item that we administered to acclimate participants to the task, and for the two-item AUT task. In particular, we obtained measures of: (a) semantic category cluster size–the average number of consecutive responses provided within a semantic category such as "buildings," excluding the first response in each cluster, (b) semantic category switching–the number of times participants changed from generating responses in one semantic category such as "buildings" to a different semantic category such as "education," and (c) semantic category revisiting–a less frequently assessed aspect of patterns of semantic processing, defined as the number of times within a given AUT test item (e.g., "cup") a participant revisited (i.e., returned) to a semantic category for which they had earlier generated one or more responses. We then examined the correlations of these content-based flexibility and persistence measures to our process-based Self-Guided Transitions. Semantic category cluster size showed no clear relation to SGTs. In contrast, semantic category switching was significantly positively correlated with AUT shift-count for the single item AUT (*r* = .36, *p* < .01) and with AUT dwell-length for the two-item AUT (*r* = .44, *p* < .01). Additionally, semantic category revisiting was positively correlated with AUT shift-count for the single item AUT (*r* = .39, *p* < .01) and with AUT dwell-length for the two-item AUT (*r* = .49, *p* < .01).

### Lab-based creativity tasks

We next sought to assess the construct validity of the various creativity tasks by examining the within-task and across-task correlations of fluency, originality, and category/semantic/reconstructive flexibility. As expected, there were predominantly strong and significant *within-task* correlations for each of the creativity tasks. Considering especially the correlations between fluency and originality, the correlations were: for AUT, *r* = .79, *p* < .01; for Conceptual Combination, *r* = .64, *p* < .01; for Suppose, *r* = .42, *p* < .01; for Figural Interpretation Quest, *r* = .19, *p* = .09. For the two lab-based tasks for which we also scored flexibility, there were likewise positive correlations between fluency and flexibility: for AUT fluency with semantic flexibility, *r* = .74, *p* < .01; for AUT fluency with reconstructive flexibility, *r* = .53, *p* < .01, and for Figural Interpretation Quest fluency and flexibility, *r* = .84, *p* < .01. Across the four lab-based creativity tasks, for each creativity dimension there were also mostly strong correlations (e.g., for fluency, *r*_AUT-Suppose_ = .61, *p* < .01; *r*_AUT-FIQ_ = .53, *p* < .01; *r*_Suppose-CC_ = .65, *p* < .01; or for originality, *r*_AUT-Suppose_ = .46, *p* < .01; *r*_AUT-FIQ_ = .24, *p* = .03; *r*_Suppose-CC_ = .45, *p* < .01).

### Creativity tasks and SGTs

Having established the construct validity of our creativity task battery, we next turned to examining our hypothesized relations between our newly developed indices of cognitive flexibility with creative performance. Table 5 presents the correlations between the Self-Guided Transitions and Creative Performance Scores on the four lab-based creativity tasks. Starting with Self-Guided Transitions on the AUT task, as shown in the second column of Table 5, the AUT Shift score positively correlated with all four within-task measures of AUT fluency, originality, semantic flexibility, and reconstructive flexibility (*r* = .34, .29, .29, and .38 respectively). Within task, the AUT Dwell score positively correlated with AUT fluency and originality (*r* = .40 and .32). Across tasks, AUT Dwell significantly correlated with fluency scores on all three independently assessed creativity tasks, including FIQ (*r* = .26), CC (*r* = .26), and Suppose (*r* = .29).

**Table 5:** Correlations between the self-guided transitions and creative performance scores.

Task and Measure	AUT Shift	AUT Dwell	Anagram Shift^1^	Anagram Dwell^1^
**Alternative Uses Task (AUT)**				
fluency	.34**	.40**	-.03	.16
originality	.29*	.32**	-.23^	.26*
semantic flexibility	.29*	.21^	-.23^	.29*
reconstructive flexibility	.38**	.03	-.16	.14
**Figural Interpretation Quest (FIQ)**				
fluency	.09	.26*	.08	-.06
category flexibility	.11	.20	-.03	.03
originality	.19	-.04	.23^	-.11
**Conceptual Combination (CC)**				
fluency	.13	.26*	-.01	.25*
originality	.11	.05	.16	-.06
**Torrance Suppose**				
fluency	.15	.29*	-.20	.17
originality	.21^	.10	-.001	.12

** *p* < 0.01 (2-tailed)

* *p* < 0.05

^ 0.05 ≤ *p* < 0.10.

*N* = 66 for all correlations with the AUT and Anagram shift-dwell indices. The values reported for the AUT and Anagram are based on participants' responses during the respective two-item tasks.

^1^ Although not included as a measure of creativity, it might be noted that performance (number of correctly found English words of 4 letters or more) on the two-set Anagram task was strongly positively correlated with the within-task SGT assessment of Anagram dwell-length, *r* = .60, *p* < .001, and not associated with Anagram shift-count, *r* = .04.

Given these findings pointing to positive associations between AUT Shift and AUT Dwell for the individual creativity tasks, in order to condense and stabilize the data, we next obtained an across-task composite measure of fluency, and an across-task composite measure of originality for the three *independently assessed* creativity tasks (that is, excluding AUT). These across-task composite measures were obtained by first z-scoring the relevant measures for each task (e.g., FIQ-fluency, CC-fluency, and Suppose-fluency, or FIQ-originality, CC-originality, and Suppose-originality), and then averaging the z-scores, yielding what we will term "Fluency3" and "Originality3" respectively. Then, to test how well SGTs conjointly predicted these across-task creativity performance measures, we performed two separate multiple linear regressions, entering AUT Shift and AUT Dwell as predictors for (1) Fluency3 and (2) Originality3.

The regression model for Fluency3 was significant, *F*(2, 63) = 11.84, *p* < .001, *R*^*2*^ = .27. Both the shift count and dwell length SGT measures explained a significant proportion of variance in Fluency3: β = .51, *t*(63) = 3.86, *p* < .001 for AUT Shift, and β = .61, *t*(63) = 4.65, *p* < .001 for AUT Dwell. The partial correlations for both AUT Shift and AUT Dwell were numerically medium-to-large (*r*_AUTshift_Fluency3_ = .44, *r*_AUTdwell_Fluency3_ = .51) and both partial correlations were numerically stronger than the zero-order correlations with Fluency3 (*r*(66) = .16 for AUT Shift, *r*(66) = .32, for AUT Dwell).

The regression model for Originality3 was also significant, *F*(2, 63) = 3.62, *p* = .03, *R*^*2*^ = .10. Paralleling the outcomes observed for Fluency3, both the shift count and dwell length SGT measures explained a significant proportion of variance in Originality3: β = .38, *t*(63) = 2.59, *p* = .01 for AUT Shift, and β = .30, *t*(63) = 2.08, *p* = .04 for AUT Dwell. The partial correlations for both AUT Shift and AUT Dwell were medium-to-small (*r*_AUTshift_Fluency3_ = .31, *r*_AUTdwell_Fluency3_ = .25) and both partial correlations were numerically stronger than the zero-order correlations with Originality3 (*r*(66) = .20 for AUT Shift, *r*(66) = .09 for AUT Dwell).

We next turned to examining if and how SGTs on the more convergent Anagram task related to creative performance (see the last two columns in Table 5). In contrast to the patterns found for the AUT, self-guided transitions on the Anagram task showed a different pattern. Whereas Shift score on the Anagram task was not robustly correlated with any of the creativity performance measures, Anagram Dwell significantly correlated with AUT originality (*r* = .26) and AUT semantic flexibility (*r* = .29), and also with CC fluency (*r* = .25).

Largely paralleling the analysis approach taken for the AUT SGTs, we next obtained an across-task composite measure of fluency, and an across-task composite measure of originality. However, we now included four independently assessed lab-based creativity tasks, that is, we included AUT, because now SGTs were separately assessed in the Anagram task. These across-task composite measures were obtained by first z-scoring the relevant measures for each task (e.g., AUT-fluency, FIQ-fluency, CC-fluency, and Suppose-fluency, or AUT-originality, FIQ-originality, CC-originality, and Suppose-originality), and then averaging the z-scores, yielding what we will term "Fluency4" and "Originality4" respectively. Then, to test how well SGTs conjointly predicted these across-task creativity performance measures, we performed two separate multiple linear regressions entering Anagram Shift and Anagram Dwell as predictors for (1) Fluency4 and (2) Originality4. These analyses revealed that, although Anagram Dwell was trending toward a positive correlation with Fluency4, *r*(66) = .20, *p* = .053 and also with Originality4, *r*(66) = .19, *p* = .066, neither the regression model nor individual SGT variables significantly explained variance in Fluency4, *F* < 1.4, or Originality4, *F* < 1.2.

### Garden design task and SGTs

Table 6 presents descriptive statistics for the Garden Design task, including the seven sketch scores, three transcript content scores, and four transcript transition scores. As an initial step, we examined the correlations between performance on the Garden Design task and the lab-based creativity tasks, using the composite measures of fluency and originality that combined across all four lab-based tasks (i.e., AUT, FIQ, CC, and Suppose). Garden Quality10 was positively correlated with Fluency4, *r*(80) = .22, *p* = .047 and with Originality4, *r*(80) = .34, *p* = .002. We also examined, in a linear regression analysis, if the three independently assessed Garden transition scores (Garden shift, Garden dwell ideation, and Garden dwell evaluation) together predicted the overall within-task creativity score of Garden Quality10. This model was significant, *F*(3, 75) = 5.46, *p* = .002, *R*^*2*^ = .18. Both Garden shift, β = .44, *t*(75) = 3.59, *p* = .001, and Garden dwell ideation, β = .37, *t*(75) = 2.97, *p* = .004, significantly contributed to the model.

**Table 6:** Descriptive statistics for the garden design task scores.

	Measures	Mean	Std. Deviation
Sketch scores	Journey diversity	4.18	1.55
	Originality	2.64	1.38
	Structure	2.26	0.68
	Elaboration	2.33	0.52
	Abstract	1.54	0.64
	Scale	1.17	0.73
	Budget	0.31	0.60
Transcript content scores	Idea unit count	76.01	31.36
	Ideas category sum	163.52	52.82
	Category sum	50.47	25.07
Transcript transition scores	Garden shift	27.66	17.00
	Garden dwell ideation	13.36	14.59
	Garden dwell evaluation	1.58	0.50

N = 81 for sketch scores, N = 80 for transcript scores and most transition counts, N = 79 for transition count dwell evaluation.

We next performed two multiple linear regression analyses with (1) AUT SGTs and (2) Anagram SGTs as predictors of the composite Garden Quality10 as the dependent variable. The first analysis, with AUT Shift and AUT Dwell entered as predictors, was not significant, *F*(2, 62) = 2.23, *p* = .12, *R*^*2*^ = .07, though the predictive power of AUT Shift on Quality10 reached significance in this model, β = .31, *t*(62) = 2.06, *p* = .04. The second analysis, with Anagram Shift and Anagram Dwell entered as predictors of Quality10 was not significant, *F*(2, 62) = 2.95, *p* = .06, R^2^ = .01, and moreover the predictive power of Anagram Shift and Anagram Dwell was not significant.

We also examined if shifting measures in the Garden Design task (the shift between thinking modes of ideation and evaluation) separately correlated with between-item shifting on the AUT and between-set shifting on the Anagram task, or if dwelling measures on the Garden Design task (dwell ideation or dwell evaluation) separately correlated with dwelling on the AUT and Anagram tasks. None of these pairwise correlations were significant, all correlations between–.11 and .17, all *p* > .17.

### Metacognition questionnaire

Our final set of analyses focused on participants' retrospective metacognitive reports of the cognitive and motivational factors contributing to their shifting and dwelling behavior during the two-item Alternative Uses and two-item Anagram tasks. S2 Data includes tables that summarize the within-method (self-report to self-report) correlations of the metacognition subscales with each other (Table A in S2 Data), and the correlations of the metacognition subscales with participants' behavior, including their self-guided transitions and the two-item task performance outcomes (Table B in S2 Data). Briefly, participants' self-reports of the various cognitive-motivational factors contributing to their own switching behavior were generally positively intercorrelated, both within the AUT (pairwise correlations between .27 and .39 for notice switch, intentionally switch, switch when stuck, switch for new, switch helped, and enjoy/challenge) and within the Anagram task (correlations between .24 and .35). Likewise, when looking across the corresponding subscales for the AUT and Anagram task (for example "notice switch" for AUT and "notice switch" for Anagram), metacognitive responses for all 7 subscales were positively correlated with each other across the two different task contexts (correlations between .26 and .63).

Looking at the relations between participants' retrospective metacognitive reports of their SGTs and their actual SGTs, for the AUT, participants' shift count was negatively correlated with their self-reported tendency to dwell for the easier item (*r* = –.27, *p* = .03). Additionally, for the AUT, participants' dwell length was negatively correlated with their self-reported noticing of switching (*r* = –.33, *p* < .01). No significant correlations were observed between Anagram metacognition and participants' SGTs on the Anagram task.

Considering the relation between participants' metacognition responses and their actual performance on the corresponding two-item tasks, there were no significant correlations between metacognition responses for the AUT and AUT performance scores. However, individuals' metacognition responses did relate to their performance on the more structured anagram task. Specifically, the total number of correctly found words on the Anagram task was positively correlated with self-reported tendencies to shift when stuck (*r* = .32, *p* < .01), and to shift for new (*r* = .23, *p* = .049), and also positively correlated with finding the two-set Anagram more enjoyable than the one-set Anagram (*r* = .23, *p* = .04). Thus, in general, participants' retrospective self-reports of the cognitive and motivational factors that contributed to their shifting and dwelling behavior on the AUT and Anagram tasks were positively correlated with each other, both within each task and across the two task contexts. However, whereas there were some suggestive associations between self-reported metacognition and participants' actual shifting and dwelling during the AUT task (that is, their cognitive search processes), for the Anagram task metacognition appeared to be somewhat more related to their task performance (that is, problem-solving outcome).

## Discussion

The aim of this study was to examine how cognitive flexibility dynamically shapes creative thinking and action. To more naturalistically operationalize the construct of cognitive flexibility, we developed and tested process-based measures of Self-Guided Transitions (SGTs) and examined their relation to creative outcomes on several lab-based creativity tasks and a more complex and extended "Garden Design" task. The SGTs assessed an individual's tendency, on a two-item task with the same general task goal, to persist in working on one of two given items and their tendency to switch to a different item. We hypothesized that self-guided transitions would predominantly reflect participants' receptive attunement to their own unfolding progress toward their task goals [26, 29, 33, 34], signaling whether they should persist (dwell) in their current direction, or instead take an alternative route (shift), indicating flexible cognitive control, thereby boosting creative performance. Given evidence that creative performance is associated with the interlinked contributions of both flexibility and persistence [2, 35], and divergent and convergent thinking [5], we hypothesized that both shifting and dwelling together would predict creative outcomes across tasks.

Collectively, our results point to the differential and combined value of our newly developed shift-count and dwell-length measures in charting creative performance and creativity-related adaptivity. Specifically, we contrasted the extent to which SGTs related to creative performance where SGTs were assessed in two different task contexts–one primarily drawing on divergent thinking (the Alternative Uses Task), the other predominantly calling on convergent thinking (an Anagram Task). We report four major findings. First and most notably, dwell length and shift count from the divergent Alternative Uses task were separately predictive of different aspects of creative performance, and together significantly predicted creative originality and fluency on a combined measure of three different, independently assessed, conceptual-perceptual tasks (Figural Interpretion Quest, Conceptual Combination and the Torrance Suppose task). Second, neither dwell length nor shift count from the convergent Anagram task were significantly predictive of across-task composite measures of fluency or originality, and showed a less systematic pattern with individual creativity measures. Third, the composite measures of fluency and originality across four lab-based creativity tasks (the three conceptual-perceptual tasks plus Alternative Uses) were significantly correlated with an overall assessment of quality in the Garden Design task, highlighting similarities between simpler lab-based creativity tasks and somewhat more extended design endeavors. Additionally, paralleling earlier studies using technically-demanding design briefs, transitions across different cognitive processes during the Garden Design task significantly predicted overall Garden Design quality. Fourth, in general, SGTs appeared to uniquely capture different aspects of cognitive flexibility than were captured by transitions within the Garden Design task. We next discuss and contextualize each of these findings.

### Self-guided transitions in different task contexts

That creative processes are best measured by a *combination* of both AUT shift count and AUT dwell length is strongly supported by our multiple regression findings. For the AUT two-item task, both the shift count and dwell length SGT measures explained a significant proportion of variance in our composite measures of creative performance on three different (independently assessed) creativity tasks, including both Fluency3, and Originality3. Tentatively, the combined contribution of switch count and dwell length coheres with theoretical accounts that creative performance depends not only on flexibility or exploration, but also on persistence or exploitation, as well as the timing, duration, and contextual-appropriateness of each [1, 41]. Cognitive science research has largely focused on the relative costs and benefits of *switching* between tasks. In contrast, our findings underscore the need to concurrently take into account factors, such as the task context, that may adaptively (or maladaptively) encourage not only switching, but *also* factors that may adaptively (or maladaptively) promote dwelling.

The measure of average dwell length may indicate an individual's dynamic modulation of choosing to persist based on their holistic sense of their goal progress and the value of continuing their current trajectory. An appropriate dwell time may facilitate creativity by allowing sufficient time for ideas to emerge or for realizing associations between ideas [2, 42, 43]. For example, extending their AUT dwell length may have allowed participants an opportunity to explore more deeply or widely the possible alternative uses of one object. This then correlated with a similarly productive exploration when these individuals were given different starting points or stimulus cues for their creative mental search, for example, when presented with an ambiguous visual shape for the Figural Interpretion Quest vs. a hypothetical scenario in the Torrance Suppose task.

All participants in the current study were given the opportunity to autonomously choose where and for how long they chose to allocate their efforts on the two-item SGT tasks. What if, instead, participants were *required* to shift their attention and efforts from one item to the other, either in a more regular (alternating) or externally-cued manner? The previously noted studies using the AUT [23] and category generation tasks [24] provide insights here. Using a version of the two-item AUT task, Lu et al. [23] found that originality was higher when participants were required to switch to the other AUT stimulus after each response they generated, than if they were given discretion to choose if, and when, they would switch between the items. Likewise, using two-item versions of the sense-impression and *ad hoc* category generation tasks, Smith et al. [24] found that participants generated responses of higher originality when they were externally prompted to switch between the two items after each minute, than when they were either left free to choose when to switch, or were required to switch only once, half-way through the task.

The results from these two earlier studies closely concur with the findings that we report here, demonstrating that switching or shifting between items on a given task is not necessarily detrimental to creative idea generation on that same task, and may instead be beneficial. The central findings from Lu et al. [23], Smith et al. [24] and the current investigation agree on this point. Yet, given the findings from their studies showing that externally-prompted shifting was associated with higher novelty than internally-prompted discretionary shifting, should we also infer that externally-prompted within-task shifting is always to be preferred over internally-prompted shifting? Not necessarily. Rather, what can be inferred from the existing evidence is that (a) shifting between items during a two-item generative task can bolster the generation of novel/original ideas, and (b) *on average*, participants do not spontaneously shift as frequently as is optimal for them, and that more frequent shifts would, on average, increment the number of original responses provided.

Equally important, it remains unclear whether it is specifically the externally-based prompting that proves beneficial (for example, through off-loading the need for internal metacognitive monitoring and the need for deciding when a switch should occur) or other associated aspects that boost novel idea generation. For example, externally-based prompts might help to bypass participants' mistaken intuitions about the potential benefits of item-switching for creative performance [23]. External prompts might also act to circumvent detrimental lost idea-generation time while individuals unproductively wait to assess if additional ideas might be forthcoming. That is, during any given dwell period, when ideas are not rapidly forthcoming, individuals may need to determine whether they are currently in the moment just before "all the good ideas will come," or are, instead, in the beginning phase of an unproductive impasse. Future research should seek to better analytically assess the possible contributions of these various factors.

Our results might also be compared with the outcomes of a recent multi-pronged investigation exploring between-person and within-individual biases toward either persistence or flexibility in several different cognitive search tasks [35]. That study used measures of clustering and switching derived from within each of four different tasks (namely, AUT, a five-point dot design task, a phonological verbal fluency task, and an anagram task). The operationalization of clusters and switches was adapted to the participant-generated content of the different tasks. For instance, for the AUT, the number of similar use-related ideas successively provided comprised a "cluster," but for the dot-design task, a cluster was defined as a series of designs following a given strategy such as rotation. Examining across-task measures of clustering and switching, Mekern et al. [35] found that clustering scores did not significantly correlate between any pair of tasks, and switching positively correlated only between the AUT and dot design. Interpreting these outcomes, these researchers suggested that their findings run counter to an expectation that the tendency to explore versus exploit is largely reflective of a general "trait-bias" toward either persistence or flexibility. Rather, they suggested that the lack of generalized across-task correlations for the clustering and switching measures–taken together with their results showing that both clustering and switching were associated with task performance, though in different degrees for different tasks–might support a within-person "metacontrol adaptivity" account. In such an account, individuals are differentially biased toward clustering or switching depending on the specific task restrictions and their task-related resources, and so adaptively modulate their level of persistence vs. flexibility to the particular task demands of each task.

Two sets of our findings are in line with the general notion of contextually-modulated adaptivity [35]. First, it might be noted that although the conclusions of these researchers are based on inferences of clustering and switching from the *content-based responses* that were produced by participants, with the characterization of that "content" itself markedly differing across the various tasks, our content-based semantic category switching and revisiting scoring of the one-item AUT versus two-item AUT are also in line with the Mekern et al. [35] interpretation. We found that even when using a similar divergent-thinking task, modifications of the task restrictions and resources–that is, the opportunity to work on only one task item (e.g., "cup") or to alternate between two presented task items (e.g., "blanket" or "flashlight")–uncovered differing relations between participants' autonomous choices for persistence and flexibility. When participants were confined to working with one task item, the number of category switches and category revisiting was correlated with their tendency to choose to work on a different item (i.e., switch count). In contrast, when participants had the opportunity to allocate their efforts between two items, then the number of category switches and category revisiting was correlated with more productive search for any one item before they alternated to the other item (i.e., dwell length).

Second, the adaptivity account also coheres well with our findings using the new *process-based* measures. In particular (a) our finding that different patterns of shift count and dwell length were associated with enhanced *within-task performance* for the two-item AUT versus the two-set Anagram tasks, and (b) our finding that whereas shift count and dwell length from one task did positively correlate with some measures of originality, flexibility, and fluency on further independently-assessed creativity tasks, these patterns also differed depending on the task (two-item AUT vs. two-set Anagram) that was used to assess flexibility versus persistence.

More specifically, the divergent patterns of correlations that we observed for the two-item AUT and two-set Anagram tasks seem to underscore task-related contextual factors that may have been associated with an adaptive *attenuation* in the tendency to shift and, instead, to dwell. In particular, whereas both shift count and dwell length were significantly positively associated with *within-task* fluency and originality on the AUT, the number of correctly generated words on the Anagram task was robustly positively associated with Anagram dwell length (*r* = .60, *p* < .001) and not with Anagram shift count (*r* = .04). Additionally, in contrast to the largely null outcomes for across-task patterns of flexibility vs. persistence reported by Mekern and colleagues [35], we found a significant positive correlation between *dwell-length* for the two-item AUT and the two-set Anagram (*r* = .31, *p* = .011). This correlation between dwell-lengths on the two (quite different) tasks perhaps points to adaptive persistence that was modulated across the different task contexts, with *both* shifting and dwelling beneficial for the AUT, but somewhat curtailed amounts of shifting combined with extended dwelling beneficial for the more tightly constrained and highly stimulus-driven Anagram task. For instance, performance on the relatively less structured open-ended AUT may have benefited from periods of both focused and defocused attention, with the latter allowing imminent associative processes to emerge into awareness [43]. In contrast, performance on the more stringently structured Anagram task, with its many working memory demands and stimulus-related constraints, may have benefited from more sustained focused attention [44].

The divergent patterns found for the correlations between the AUT versus Anagram Self-Guided Transitions to the different across-task composite creativity measures might similarly be accommodated by an *adaptivity* account that *concurrently* takes into account both shift count and dwell length and the particular demands of each task on which flexibility and persistence are assessed. For instance, whereas AUT shift count on its own did not significantly correlate with our composite across-task measures of Originality (Originality3, based on composite originality scores for the Figural Interpretation Quest, Conceptual Combination, and Torrance Suppose tasks), incorporating *both* AUT-shift and AUT-dwell in the multiple regression analyses revealed that each measure individually accounted for a significant proportion of variance (β of .38 for AUT-shift and β of .30 for AUT-dwell). A somewhat similar, but less pronounced and weaker statistical pattern was found for the combination of AUT-shift and AUT-dwell in relation to the composite quality measure for the more complex Garden Design task.

In marked contrast, Anagram-shift tended to be a negative correlate of Garden Quality10 when incorporated along with Anagram-dwell into the regression analyses. Contrasting the different amounts of information given in the stimuli for the two-item AUT versus the two-set Anagram task, whereas shifting between "blanket" versus "flashlight" might occur quite readily with minimal cognitive effort, shifting between one set of nine letters, and another set, and monitoring which words had already been found for each set might require more extensive updating of working memory and impose a greater risk of cross-item interference.

### Self-guided transitions in relation to complex creative tasks

A unique contribution of the current work is the bridging between research and theory in cognitive neuroscience on one side, and design studies on the other, through the inclusion of process (transition) and outcome assessments across several laboratory-based creativity tasks *and also* in the more extended, naturalistic Garden Design task. Especially notable outcomes from this more comprehensive multi-task investigation of creativity were that the global assessment of performance on the naturalistic design task (Garden Quality10) significantly positively correlated with our composite measures of fluency and originality based on four different lab-based creativity tasks (i.e., Fluency4 and Originality4). We also found–in line with results from more complex design-based protocols such as that of Atman et al. [4]–that within-task transitions on the Garden Design task significantly correlated with the overall global quality score. This finding suggests that within-task transitioning between idea generation and idea evaluation is similarly important for creative performance on a somewhat briefer, more accessible task and for the more temporally-extended, technically-demanding complex design tasks used in earlier design studies research. There were, however, few correlations between the transitions measures on the two-item tasks and the transition measures on the Garden Design task, although the regression analysis intimated that the AUT shift count was modestly predictive of overall Garden Design quality performance.

Taken together, these findings suggest that there are similarities in the underlying factors that contribute to generating original and novel ideas in different tasks, for example, as demonstrated by the significant positive correlation between the multi-task assessment of lab-based creativity and overall quality of Garden design. Yet, there may be different factors that underpin when and why individuals choose to transition between different cognitive processes within a single complex extended creative task (e.g., generating ideas and evaluating those ideas) versus what prompts them to transition between working on one of two possible items within a relatively simple task. Whereas we tabulated movements between ideation and evaluation in the Garden Design Task, what we measured in the two-item AUT task is movements of attention and effort allocation from one item to another. Although an individual's successive responses during the AUT might in some cases be conceptually or perceptually linked to one another, the task requirements do not demand such connections. Successive AUT responses can be independent of one another. In contrast, during the Garden Design task participants needed to consider the interrelations between the various ideas they had and would generate. In other words, our SGTs capture the choice to work on different items with similar goals, whereas Garden transitions reflect mental movements across different thinking modes to accomplish a single overarching goal. Nonetheless, given that AUT shift count appeared to be modestly predictive of overall Garden Design quality, the cognitive flexibility indexed by SGTs on a simpler creative task may contribute to creative outcomes on a more complex task.

### Metacognition and choosing to shift or to dwell

Researchers exploring voluntary or spontaneous task switching have speculated about the cognitive-motivational factors that might prompt participants to switch or to stay, such as top-down and deliberately chosen switching versus more bottom-up spontaneous fluctuations in one's inner psychological and physiological state [16]. We attempted to examine some of these factors with our retrospectively administered assessments of participants' metacognitive experience during the two-item choice tasks. These questionnaires revealed that there were some associations between participants' self-awareness and their shift-dwell behavior, and appeared to indicate across-task parallels in their metacognitive perceptions of their task process. For example, participants' self-reports regarding particular cognitive-motivational factors (e.g., whether they intentionally switched, or switched to experience something new, or switched because they were stuck) were generally positively intercorrelated, both within the AUT and within the Anagram task, and across the corresponding subscales for the two different task contexts.

However, whereas participants' metacognitive reports tended to correlate with their actual dwelling and shifting behaviors for the AUT (process), for the Anagram task they tended to correlate with the number of correct responses they had generated (outcome). One possibility is that these divergent patterns (correlations with process vs. outcome) in the two different task contexts relate to the aforementioned different demands on working memory for tracking ongoing progress during the Anagram task versus the AUT. A second possibility is that stronger and more consistent correlations between metacognition and both process and outcome behavior would be observed if the metacognitive assessment was altered. Rather than asking participants to indicate how much they agreed with various characterizations of their motivation, they might be asked to more directly estimate how often they engaged in specific actions (e.g., the number of times they switched when stuck, or the number of times they switched because they wanted to work on something new).

Our across-task correlations indicated that dwell length for the AUT and Anagram task was significantly positively correlated, but there was no across-task correlation for shift count. Does this point to different cognitive-motivational factors underlying the choice to shift in these two different task contexts? Participants who reported less often noticing their switching behavior on the AUT, on average had longer AUT dwell lengths and, conversely, participants who reported that they chose to continue working on an easier AUT item had lower average AUT shift counts. These findings suggest that participants were, at least to some extent, able to recollect and assess the cognitive-motivational impetus for their choices to shift or dwell on the two-item AUT and two-set Anagram tasks and that these recollections to some degree corresponded with their actual behavior. Still, it is possible that, because the assessments were given only after completion of both the AUT and Anagram tasks, the intervening delay may have attenuated participants' specific recollection of how and why they chose to shift or dwell.

There is a trade-off between adopting retrospective versus in-the-moment (concurrent) probes of the cognitive-motivational factors underpinning participants' choices to shift between items or to continue working on the same item. Whereas concurrent probes of participants' metacognitive experience during the two-item tasks would have the benefit of mitigating demands on memory, such in-task probes might impose important drawbacks of reactively changing the spontaneous (autonomous) task processes that are of central interest. Nonetheless, concurrent probes might help to adjudicate between different reasons that participants choose to switch from one item to another. For example, brief interludes away from a specific task may help an individual to "forget" fixation (move past a fixated idea), and/or offer the fortuitous opportunity for assimilation of new information that sparks fresh directions of thought, and/or allow time for "unconscious work" [24, 41, 45, 46].

### Future directions, limitations, and conclusion

Despite the many promising findings revealed by this study, there are many directions for further exploration. Although we examined Self-Guided Transitions in two quite different task settings representing both predominantly divergent-based (AUT) and convergent-based (Anagram) task goals, it remains an open question of how well our dwell-length and shift-count measures generalize to variants of the current tasks, to other types of two-item self-guided choice tasks, or to other creativity tasks. For example, a new self-guided choice task might include two Figural Interpretation Quest items, or creativity tasks that rely more heavily on convergent thinking processes might also be tested. Also, as an initial step, each of our SGT tasks involved two task items. To further explore individuals' internal and external cognitive search patterns and autonomous choice in an even richer context, future research might include three or more task items. Inclusion of three or more task items would additionally help to better separately characterize the choice to dwell and the choice to shift, attenuating the negative correlation between shift-count and dwell-length.

Additionally, although a marked strength of the current study is that we included multiple and varied assessments of creative performance, in order to avoid overburdening participants we did not include assessments of control variables (such as general mental ability or vocabulary) that might contribute to creativity [47], and we also did not include longer-term trait-based rather than state-based assessments of creative activities or achievements [48]. For instance, are the individuals who show especially high levels of creative adaptivity on lab-based measures–as assessed by Self-Guided Transitions–also likely to show higher levels of innovative and original thinking in contexts beyond the lab? Is there a shared form of metacognitive awareness or sensitivity that aptly guides individuals in their creative attentional and effort allocations during comparatively shorter periods of time, such as those we examined in the current study, and during more prolonged creative endeavors, that might span days, weeks, months, or even years? These open questions themselves underscore the creative generative potential of our newly introduced Self-Guided Transition measures, that are comparatively straightforward, non-inferential, and can be unobtrusively obtained as individuals naturally and spontaneously allocate their time and effort to one of two presented items within a single task.

In conclusion, we have demonstrated the value of *conjointly considering* an individual's autonomous choices to either continue devoting their attention and effort to working on a current problem, or switching instead to working on a similar item, as a new process-related measure of creative adaptivity. Notably, the combined dwell length and switch count measures from the two-item divergent thinking task were significantly predictive of originality (and fluency) on a composite measure of three other (independently assessed) lab-based creativity tasks, and also of the overall quality of a more complex design problem. Thus, this Self-Guided Transitions paradigm offers an especially promising approach to operationalize creativity-related cognitive flexibility. Self-Guided Transitions offer a new means to assess the interaction of contextual and person-related factors that contribute to alterations in dynamic shifts between persistence versus varying, and how the timing, sequencing, and duration of such shifts influence different aspects of creative performance.

## Supporting information

S1 Data Creativity task and garden design scoring details. (PDF)

S2 Data Metacognition questionnaires. (PDF)

S3 Data Comparison of scoring methods. (PDF)
