# Supplementary material for: Charting the contributions of cognitive flexibility to creativity: Self-guided transitions as a process-based index of creativity-related adaptivity
Source: PLoS One. 2020 Jun 11;15(6):e0234473. doi: 10.1371/journal.pone.0234473 (PMC7292033; doi:10.1371/journal.pone.0234473)
Supplement: S1 Data — (PDF) [file pone.0234473.s001.pdf]

S1 Supporting Information: Creativity Task and Garden Design Scoring Details

Charting the contributions of cognitive flexibility to creativity:
Self-Guided Transitions as a process-based index of creativity-related adaptivity

Yihan Wu, Wilma Koutstaal

Interrater reliability

The interrater reliability for the various lab-based creativity measures ranged between .78 and 1.00. Given the high reliability, we averaged the two raters' scores. For the Garden Design task, the average interrater reliability across the 7 sketch scores was .78, so we also used the average of the two raters' scores. Two raters separately coded the Garden Design think-aloud transcripts, with both raters coding 22.5% of the transcripts. They achieved interrater reliability of .87; for the transcript measures, the scores are based on one of the two raters.

Garden Design Task Sketch score subscale

(1) Journey diversity. The journey diversity index reflected how well the sketched plan met the requirement that the garden should provide a journey or a series of experiences for the visitors who walk in it. The ratings were made on a 7-point scale. The sketches were given 1 point if there was only one type of experience involved, 2 points if there were two types of experience, and so on. Sketches that involved seven types of experience or more were given 7 points. Different types of experience can refer to different types of items presented (e.g., plants, animals), different sensory modalities that were evoked (e.g., smell, sound), and/or different activities visitors may engage in (e.g., picking strawberries, visiting a butterfly house).

(2) Originality. The originality index reflected how innovative a design was on a 7-point scale. Spatial layouts (e.g., the shape of the garden, layout of the path), metaphorical/analogical thinking, uniqueness of the theme, and the presence of uncommon objects or activities were considered while rating. The uniqueness of the theme and the presence of uncommon objects aspects can be scored from 0 to 2, depending on how unique the theme is and how uncommon the objects are respectively. The other aspects can be scored from 0 to 1. The originality scores are the sum of the above listed 5 aspects.

(3) Elaboration. The elaboration index refers to the level of details expressed through pictorial or verbal illustrations on a 3-point scale. If the majority of the elements across different objects and activities were described in detail, the design would be rated as highly elaborated. If some elements were described in detail but only limited to certain types of elements or the detailed descriptions were repetitive, the design would be rated as intermediately elaborated. If the sketch was mostly schematic with few details, it would be rated as low in elaboration.

(4) Structure. The structure index refers to how well the design was planned overall on a 3-point scale. Whether there was(were) well-defined path(s), a clear entrance and/or exit, and reasonably separated sections were considered while rating.

(5) Abstraction. The abstraction index reflects how abstract the ideas presented in the sketches were on a 3-point scale. If the theme or elements of the design were not just concretely associated with gardens, the sketch would be rated as high in abstraction. For example, adesign with the theme of life, or a garden including statues, murals, or other representations of influential people or places, was considered more abstract than a design filled with trees and flowers. The sketches that reflect designers' consideration of an extended temporal period, such as a design that included an irrigation system or a donation box indicating an awareness of how plants grow and how the garden runs sustainably, were also viewed as more abstract.

(6) Scale. The instructions to participants indicated that their garden should be between 10 x 10 yards to 10 x 22 yards, and that they should indicate the scale they had used on their sketch. Whether participants considered the scale of the garden was recorded: 0 for not mentioned, 1 for explicitly mentioned, and 2 for detailed and explicit consideration.

(7) Budget. The instructions to participants indicated that they had a budget of up to $100,000 for their garden. Similar to the scoring method for scale, *w*hether participants considered the budget constraints was assessed: 0 for not mentioned, 1 for explicitly mentioned, and 2 for detailed and explicit consideration.

Garden Design Task transcript content coding

The Garden Design task transcripts were segmented into idea units. Each idea unit was then coded into one or more of 218 idea-content and cognitive process-related categories (31 primary categories and 187 subcategories). These idea categories covered both the variety in the content of participant's design ideas (e.g., plants, water features) and different types of cognitive activities (e.g., positive or negative evaluations) involved during their design process. The subtypes encoded the different aspects (e.g., plant subtype, location in the garden) of the objects and activities participants focused on during the idea units.

Garden Design Task transcript transition score

The coded idea units were further grouped into two thinking-mode groups: ideation and evaluation. Two raters counted the shifts between ideation and evaluation stages for the Garden shift count. Average dwelling lengths were calculated both within each type of design thinking mode (i.e., Garden dwell ideation, Garden dwell evaluation) and across the thinking modes (i.e., Garden dwell both).
