# Supplementary material for: Charting the contributions of cognitive flexibility to creativity: Self-guided transitions as a process-based index of creativity-related adaptivity
Source: PLoS One. 2020 Jun 11;15(6):e0234473. doi: 10.1371/journal.pone.0234473 (PMC7292033; doi:10.1371/journal.pone.0234473)
Supplement: S2 Data — (PDF) [file pone.0234473.s002.pdf]

S2 Supporting Information: Metacognition Questionnaires

Charting the contributions of cognitive flexibility to creativity:
Self-Guided Transitions as a process-based index of creativity-related adaptivity

Yihan Wu, Wilma Koutstaal

List of Metacognition Subscales

Enjoyable: question 1 (reverse-scored) + question 2
Choose dwell for easier: question 3
Notice switch: question 4
Intentionally switch: question 5
Switch when stuck: question 6
Switch for new: question 7
Switch helped: question 8 + question 9 (reversed)

List of Metacognition Questions

Questions are stated in the Anagram version. The text was identical for the AUT questions except "set" was replaced with "object". Subscales are indicated in parentheses.

1. Compared with the one-set task, I found the two-set task was more challenging. [Enjoyable, reverse-scored]
2. Compared with the one-set task, I found the two-set task was more enjoyable. [Enjoyable]
3. I found one set was easier to generate uses for than the other set, so I stayed with the easier set longer. [Choose dwell for easier]
4. During the task, I noticed that I was switching between the two sets. [Notice switch]
5. During the task, I intentionally switched between the two sets. [Intentionally switch]
6. I switched from one set to the other when I could not think of solutions for the current set. [Switch when stuck]
7. I switched when I wanted to work on something different or new. [Switch for new]
8. I found switching between the two sets helped me to think of solutions. [Switch helped]
9. I found switching between the two sets was interruptive. [Switch helped, reverse-scored]

Analysis Note

All of the items were answered on a 5-point Likert-scale (1 = Strongly disagree, 2 = Disagree, 3 = Neutral, 4 = Agree, 5 = Strongly agree); additionally, participants were given the option of indicating that the item was not applicable (6 = Not applicable). Answers of "Not applicable" were coded as no response except if a "nonapplicable" response was given for an item that was part of a two-item subscale, and the other item on the scale was answered, then the missing response was replaced with the inferred value from the answered item.

Table A. Within-Method (Self-Report to Self-Report) Metacognition Correlations.

Metacognition responses regarding the AUT (within-questionnaire correlations)

Notice switch - Intentionally switch, $r = .27$*
Notice switch - Switch when stuck, $r = .31$**
Notice switch - Switch for new, $r = .34$**
Notice switch - Switch helped, $r = .35$**
Intentionally switched - Switch when stuck, $r = .32$**
Switch when stuck - Switch for new, $r = .27$*
Switch helped - Enjoy/challenge, $r = .39$**

Metacognition responses regarding the Anagram task (within-questionnaire correlations)

Notice switch - Switch when stuck, $r = .35$**
Intentionally switched - Switch when stuck, $r = .24$*
Switch when stuck - Switch for new, $r = .26$*
Switch helped - Enjoy/challenge, $r = .32$**
Choose dwell for easier - Enjoy/challenge, $r = –.29$**
Choose dwell for easier - Switch helped, $r = –.27$*

Metacognition responses for the AUT with Metacognition responses for the Anagram task (across-questionnaire correlations for matched/corresponding items only)

Enjoy/challenge AUT - Enjoy/challenge Anagram, $r = .28$*
Choose dwell for easier AUT - Choose dwell for easier Anagram, $r = .26$*
Notice switch AUT - Notice switch Anagram, $r = .63$**
Intentionally switch AUT - Intentionally switch AUT, $r = .53$**
Switch when stuck AUT - Switch when stuck Anagram, $r = .60$**
Switch for new AUT - Switch for new Anagram, $r = .44$**
Switch helped AUT - Switch helped Anagram, $r = .56$**

[a] Given missing data, the number of observations for the reported correlations ranged between 68 and 81.
** $p < .01$, * $p < .05$, ^ $p < .10$

Table B. Correlations of Meta-Cognition Responses with Self-Guided Transitions and Performance Measures [a].

Metacognition responses with Self-Guided Transitions

<u>*AUT*</u>
Choose dwell for easier - AUT shift count, $r = -.27$*
Notice switch AUT - AUT dwell length, $r = -.33$**

<u>*Anagram*</u>
Intentionally switch Anagram - Anagram shift count, $r = .23$^
Switch helped Anagram - Anagram shift count, $r = .24$^

Metacognition responses with Within-Task Performance

<u>*AUT*</u>
Nonapplicable [b]

<u>*Anagram*</u>
Enjoy/challenge Anagram - Anagram total correct responses, $r = .23$*
Switch when stuck Anagram - Anagram total correct responses, $r = .32$**
Switch for new Anagram - Anagram total correct responses, $r = .23$*

---

[a] Given missing data, the number of observations for the reported correlations ranged between 60 and 81.
** $p < .01$, * $p < .05$, ^ $p < .10$.
[b] There were no significant correlations between metacognition responses for the AUT and AUT performance scores.
