# Supplementary material for: Charting the contributions of cognitive flexibility to creativity: Self-guided transitions as a process-based index of creativity-related adaptivity
Source: PLoS One. 2020 Jun 11;15(6):e0234473. doi: 10.1371/journal.pone.0234473 (PMC7292033; doi:10.1371/journal.pone.0234473)
Supplement: S3 Data — (PDF) [file pone.0234473.s003.pdf]

S3 Supporting Information: Comparison of Scoring Methods

Charting the contributions of cognitive flexibility to creativity:
Self-Guided Transitions as a process-based index of creativity-related adaptivity

Yihan Wu, Wilma Koutstaal

Given the theoretical and methodological novelty of our Self-Guided Transition (SGT) process-based measures to assessing flexibility (shift count) and persistence (dwell length), we also scored flexibility and persistence in alternative ways to offer comparisons. With these comparisons, we aim to address two sets of questions.

(I) To what extent do the semantically-based content-scoring approach to flexibility and the process-based SGTs point to similar underlying cognitive processes? Does the relation between content-based versus process-based scoring differ when there is only one item versus when participants can freely choose between working on either one or another of two items?

(II) How does creative performance relate to an alternative process-based measure of persistence? Should the first response in a consecutive series of responses or a single response to an item be excluded from the measure of persistence, or should all responses be included, as a continuous and complete tracking of participants' thinking across time?

Specifically, we additionally assessed flexibility using semantically-based content-based scoring (obtaining measures of semantic category cluster size, semantic category switching, and semantic category revisiting). Furthermore, we assessed persistence using a modified definition of dwell length in which we counted only dwell lengths of more than one response (that is, the first response in a consecutive series of responses or a single response were not counted as dwelling; this recoded measure is referred to below as "dwell-without-1").

I. Semantically-based Content-based Flexibility Measures

One rater re-scored both the one-object AUT (cup) and the two-object AUT (blanket, flashlight) using content-based scoring to obtain measures of semantic category cluster size, semantic category switching, and semantic category revisiting. The scores for the two-object AUT are averages of the scores for each object.

The content-based measures of categorical persistence and shifting were:

1. *Cluster Size:* The consecutive responses within a given semantic category (e.g., "buildings") were first recorded on the excel sheet as the raw cluster length. All non-zero cluster lengths across the two objects (blanket, flashlight) were subtracted 1 and then averaged across all the new scores as the final cluster size for each participant.
2. *Category Switch:* The number of switches to a different semantic category in time (e.g., generating a response in the category of "buildings" and then in the category of "education").

3. *Revisit Count*: The number of times a participant returns (comes back) to a semantic category for which they had earlier generated one or more responses.

Outlier Checks
No participant was a consistent outlier across all tasks, so all data were included in the following analyses.

Descriptive Statistics

Table A. Descriptive Statistics

	Mean	SD
ClusterSize_Cup	.85	1.13
ClusterSize_Avg2	1.15	.90
CategorySwitch_Cup	5.88	2.83
CategorySwitch_Avg2	4.83	2.00
Revisit_Cup	1.27	1.44
Revisit_Avg2	1.22	.94

N = 81. Measures for the one-object task are designated with "Cup," measures for the two-object task are designated as "Avg2." For participant 135 and 125, no responses were given for the AUT task object "flashlight." Thus, the final scores for the two-object condition for these two participants were the scores for the task object "blanket" instead of the average scores for both "flashlight" and "blanket."

Correlations between content-based flexibility measures for one-object and two-object AUT

The correlations across the one-object and the two-object AUT for each of the three different content-based flexibility measures were as follows:

Cluster size, $r = .43^{**}$
Category Switch, $r = .65^{**}$
Revisit, $r = .31^{**}$

The positive correlations for all three content-based flexibility measures across the two task contexts show that participants tended to use at least somewhat similar semantic-search strategies in both the one-object and two-object tasks. However, the magnitude of the correlations also suggests that there may be somewhat different cognitive and exploratory strategies invoked when participants have the option of working on either one of two objects, rather than exclusively working on one object throughout. Whereas content-based measures indicate how frequently participants moved between different semantic categories to generate responses with the same stimulus, in the two-object setting, participants might opt to work on a different task stimulus (rather than continuing to generate responses to a single object). Turning their attention to the second object might externally cue the participant to "restart" or "redirect" their search and problem-solving efforts, and thus the process of generating responses for two stimuli within the same task period was different from generating responses for a single object at a time.

Correlation between Process-based SGTs and Content-based Measures

Table B. AUT SGT and Categorical Persistence and Shift

	AUT Shift	AUT Dwell	AUT Dwell-without-1
Cluster Size Cup	.02	.09	.15
Cluster Size Avg2	.19	-.003	.13
Category Shift Cup	.36**	.14	.21^
Category Shift Avg2	.20	.44**	.41**
Revisit Cup	.39**	-.01	.18
Revisit Avg2	.09	.49**	.43**

** Correlation is significant at the 0.01 level (2-tailed). Measures for the one-object task are designated with "Cup," measures for the two-object task are designated as "Avg2."

As can be seen from Table B, the content-based flexibility score in the one-object setting (category shift cup) is positively correlated with the process-based flexibility measure (AUT shift; r = .36), again suggesting that the two approaches to assessing flexibility capture some common cognitive function. In contrast, in the two-object setting, the content-based flexibility score (category shift avg2) is positively correlated with the process-based dwelling measure (AUT dwell, r = .44). Additionally, whereas revisiting a category is positively correlated with AUT shift in the one-object setting (re-visit cup, r = .39), revisiting is correlated with AUT dwell in the two-object setting (re-visit Avg2, r = .49).

These divergent patterns again suggest that how semantic flexibility is manifested in a two-object setting is different from how it is revealed in the one-object setting. This may point to the differences in the environmental cueing provided by one versus two objects and how the environment provides support for search in semantic space.

II. Alternative Process-based Measure of Persistence

The measure of dwell length that we propose includes all written responses, including both the first response in any consecutive series of responses, and instances where participants only gave one response before switching to a different item. As noted in the manuscript, this operationalization of persistence provides a continuous tracking of participants' thinking across time even if they move to a different task item after successfully generating only a single response. However, it might be asked if providing a single response should be counted as "dwelling". By analogy, in the semantic-based approach to establishing cluster size, the first response does not count (that is, to find cluster size, all non-zero cluster lengths are first subtracted 1). To evaluate the pattern of results including how persistence relates to creative performance with this alternative operationalization of persistence, dwell-lengths were re-coded after subtracting the first response (so that only responses after the first given response were counted) and averaging over the non-zero dwell durations. As noted earlier, this recoded measure is referred to as "dwell-without-1".

Table C. Descriptive Statistics

	Mean	SD
AUT Dwell-without-1	2.61	1.72
Anagram Dwell-without-1	4.46	2.59

Three high-scoring outliers were identified for AUT Dwell-without-1: 126, 143, 158. There were no outliers for Anagram Dwell-without-1. No participant was an outlier consistently across the two tasks, and thus all participants are included in further analysis.

Table D. Correlations of SGT Dwell and Dwell-without-1 with Lab-based Creative Performance

Task and Measure	AUT Dwell	AUT Dwell-without-1	Anagram Dwell	Anagram Dwell-without-1
Alternative Uses Task (AUT)				
fluency	.40**	.46**	.16	.18
originality	.32**	.25*	.26*	.30*
semantic flexibility	.21^	.23^	.29*	.29*
reconstructive flexibility	.03	.00	.14	.11
Figural Interpretation Quest (FIQ)				
fluency	.26*	.32**	-.06	.01
category flexibility	.20	.19	.03	.09
originality	-.04	-.07	-.11	-.17
Conceptual Combination (CC)				
fluency	.26*	.33**	.25*	.30*
originality	.05	.09	-.06	-.08
Torrance Suppose				
fluency	.29*	.40**	.17	.19
originality	.10	.13	.12	.09

As can be seen from Table D, although there are slight changes in the magnitude of the correlations of dwell length versus dwell-without-1 with creative performance, the correlations are very similar.

Taking everything into consideration, so as to capture a full picture of the participant's thinking processes, we recommend using the operationalization of process-based persistence that uses all of each participant's written responses. We believe that this scoring approach for dwell length best addresses the longer-term theoretical and empirical needs of researchers of creative cognition and cognitive search by inclusively tracing participants' autonomous allocation of attention and effort to multiple task items.
